# Knowledge gaps related to HIV and condom use for preventing pregnancy: a cross-sectional study among migrants in Sweden

**DOI:** 10.1186/s12889-024-19839-2

**Published:** 2024-08-28

**Authors:** Veronika Tirado, Nicola Orsini, Susanne Strömdahl, Claudia Hanson, Anna Mia Ekström

**Affiliations:** 1 Department of Global Public Health, Karolinska Institutet, Stockholm, Sweden; 2 Department of Medical Sciences, Infectious Diseases, Uppsala University, Uppsala, Sweden; 3 Department of Disease Control, London School of Hygiene and Tropical Medicine, London, UK; 4 Department of Infectious Diseases, Venhälsan/South General Hospital, Stockholm, Sweden

**Keywords:** Contraception, Condoms, Pregnancy, HIV, Sexual and reproductive health, SRHR, Survey, Knowledge, Migrants, Sweden

## Abstract

**Background:**

Information and knowledge of sexual and reproductive health and rights (SRHR) plays a crucial role in promoting safe sexual practices among young migrants. We aimed to assess the sociodemographic factors of migrants associated with knowledge of condoms and the prevention, treatment, and transmission of HIV to highlight the need for SRHR information, including comprehensive sexual health education.

**Methods:**

A cross-sectional survey was conducted (2018–2019) among migrants at Swedish language schools and high schools across Sweden. The survey included questions about knowledge of condom use for preventing pregnancy and HIV treatment and transmission. Descriptive statistics were calculated, and multivariable logistic regression analyses were performed to assess the responses to the knowledge questions and sociodemographic characteristics.

**Results:**

Out of 3430 respondents (median age: 35, interquartile range: 20), approximately 39% were unaware that condoms can prevent unplanned pregnancies. Only 58% of the respondents knew that condoms reduce the risk of contracting HIV. About 77% were unaware of HIV treatment, and 52% reported not knowing that a woman with HIV could transmit the virus to her baby during pregnancy or breastfeeding. Incorrect knowledge about condom use to prevent unwanted pregnancy was associated with several factors: younger age (15–19 years) adjusted odds ratio (aOR) 1.35; 95% confidence interval (CI), 1.02–1.79); female respondents (aOR: 1.68; 95% CI 1.36–2.07); lack of previous sexual health education (aOR: 2.57; 95% CI 2.11–3.13); low level of education (aOR: 1.30; 95% CI 1.04–1.61). Originating from the Americas, European, or sub-Saharan African regions was associated with a 34–42% decreased likelihood of incorrect knowledge that condoms can reduce the risk of HIV infection compared to respondents from the Middle East and North Africa (MENA). More than half (64%) of respondents reported needing more SRHR information.

**Conclusions:**

We found significant knowledge gaps on HIV and condom use for preventing pregnancy among migrants in Sweden. Comprehensive sexual health education in language schools, along with information to newly arrived migrants from diverse regional backgrounds and targeted sexual health services to younger individuals, women, and those who lack sexual health education, are needed to address these information gaps and provide crucial SRHR education and information.

## Introduction

Unintended or unwanted pregnancy often stems from a lack of decision-making power, autonomy, or the lower value placed on women’s lives [1]. The inability to negotiate safer sex also makes young women particularly vulnerable to HIV. In sub-Saharan Africa (SSA), adolescent girls and young women aged 15–24 are over three times more likely to acquire HIV than their male peers, accounting for over 77% of new infections in this age group as of 2022 [2, 3]. Significant achievements have been made in the global fight against HIV and AIDS, primarily attributed to the impact of effective treatments, such as antiretroviral therapy (ART) and the prevention of mother-to-child transmission (PMTCT) for individuals living with HIV [3]. In addition, the consistent use of condoms, both male and female condoms, remains the most accessible method for preventing both unintended pregnancies and sexually transmitted infections (STIs), including HIV [4]. In Northern Europe, unintended pregnancies ending in abortion declined by 21% between 2015 and 2019 [1, 5]. Despite progress, unintended pregnancies remain a global challenge, with a notable surge during the COVID-19 pandemic, particularly in low-and middle-income countries [6]. The lack of awareness and access to comprehensive sexual health education, unsafe sexual practices, misconceptions, and inconsistent contraception use, including condoms, have been identified as root causes of unintended pregnancies and HIV acquisition among young people [2, 7].

Migrants, being a vulnerable population, face a higher risk of contracting HIV and often have reduced access to prevention and treatment services [8]. In 2022, nearly one million people sought asylum in European countries, with the largest group of applicants being from Syria, Afghanistan, and Turkey [9, 10]. Approximately 42% of newly reported HIV cases in the European Union were among migrants, with an estimated 19% of HIV diagnoses in Sweden occurring after migration, attributed to factors such as personal, structural, or socioeconomic barriers [9, 11, 12]. The migration process often disrupts the education of young migrants, limiting their access to essential sexual health information and awareness within educational or institutional settings [13, 14]. Several studies in Sweden have indicated that young migrants, due to a lack of awareness, were not fully exercising their right to access health services [15, 16]. To address these challenges, it is imperative to focus on key aspects of sexual and reproductive health and rights (SRHR) among migrant groups [17], and this includes areas such as condom use for contraception and HIV prevention, HIV treatment, and the mode of HIV transmission.

### SRHR in Sweden

The Swedish Association for Sexuality Education (*Riksförbundet för sexuell upplysning*,* RFSU*) plays a crucial role in providing comprehensive sexual health education to young people, including migrants, by offering valuable information on STIs and condom education in Sweden [18]. This education resulted in 95% of the professionals working with migrants in Sweden, as well as migrants themselves, reporting an increase in their knowledge of SRHR, sexuality and health [19]. Despite these efforts, there remains a significant lack of knowledge about HIV and condom use among migrants, which is often exacerbated by various social determinants, such as language barriers and limited education [20]. Previous research in Sweden has shed light on the challenges faced by migrants living with HIV, revealing a widespread lack of knowledge about the virus and its treatment, indicating that HIV is often under-prioritised compared to social integration [21–23]. Furthermore, migrant parents in Sweden report difficulties in reconciling their cultural norms and values from their countries of origin with those in Sweden, particularly when discussing sexual health with their children [24]. Healthcare providers have identified young migrants’ lack of awareness about contraception methods, including condoms and the prevention of STIs, as a significant barrier to addressing SRHR in Sweden [21]. While previous research in high-income countries has compared the general HIV-related knowledge of migrants and host populations, there is a limited understanding of this knowledge gap regarding HIV and condom use for preventing pregnancy among migrants in Sweden [25–27].

### Rationale and objectives

The assessment of SRHR knowledge among recently arrived migrants closely aligns with the Sustainable Development Goals (SDGs), particularly in the areas of health (SDG Goal 3), reduced inequalities, and universal access to sexual and reproductive health care, information, and education [28]. The SDGs are a global framework guiding development efforts, with Goal 3 emphasising the importance of good health and well-being for all [29]. In this context, assessing migrants’ knowledge of condoms for preventing pregnancy and HIV transmission is crucial, given the limited research on this topic among migrant populations.

Our objective was to describe and assess sociodemographic differences among recently arrived migrants and their knowledge of condoms as a contraceptive method for preventing pregnancy and HIV transmission. Additionally, we aimed to identify any information needs related to SRHR among migrants.

## Methods

### Study design and setting

**Fig. 1:**
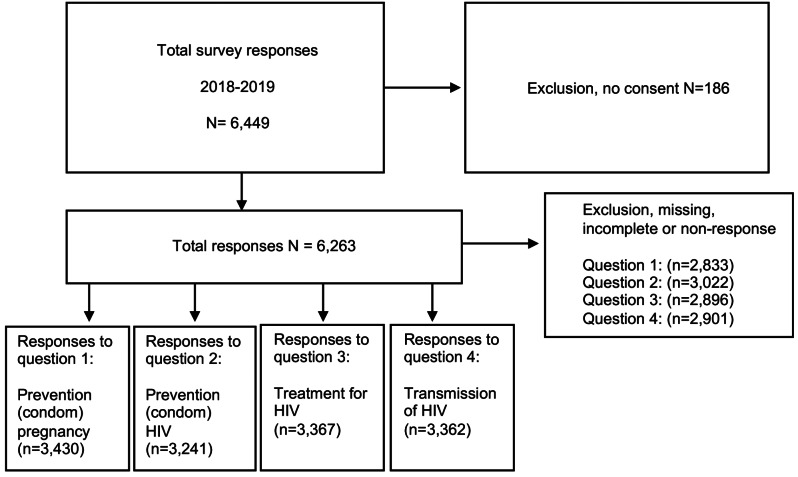
Flow chart of this study

The sample for this cross-sectional study was obtained through a self-reported online survey conducted between November 2018 and December 2019 (Fig. 1), which formed part of a previous publication on the Swedish abortion law and other SRHR-related laws [30]. The survey was administered in Swedish language schools and high school (*gymnasiet*) classrooms and was accessible through a secured website. The participants were given a link and password to complete the questionnaire anonymously using a digital device. A research team member was available on-site at the schools to address questions and ensure quality control during the data collection phase.

### Survey questions

The pre-tested survey consisted of 75 questions covering sociodemographic background and various topics, including health needs, sexual risk-taking behaviours and attitudes towards norms and values, and knowledge of HIV. This questionnaire was available to the participants in the study population’s seven most widely spoken languages (Arabic, Dari, English, Somali, Spanish, Swedish, and Tigrinya). The survey instrument underwent a comprehensive development process, including back-translating the questions and pre-testing the survey to ensure clarity, relevance, and cultural appropriateness, thereby minimising potential response biases. Our analysis focused on the three questions and responses concerning HIV prevention, treatment, and transmission, and these questions were formulated and adapted using the Living with HIV survey in Sweden, carried out previously by members of our team on behalf of the Public Health Agency of Sweden [31]. We also included one question related to condoms, which was formulated in line with the SRHR definition provided by the Guttmacher-Lancet Commission [17].

### Participants and eligibility criteria

The International Organization of Migration defines a migrant as “a person who moves away from his or her place of usual residence, whether within a country or across an international border, temporary or permanently and for a variety of reasons,” such as violence, conflict, natural disasters, and human rights abuse [32]. The inclusion criteria were migrants who were foreign-born and had migrated to Sweden. The eligible participants were individuals aged 15 years and older who provided their consent. We excluded individuals who did not meet the inclusion criteria and those who could not consent to participate in the study.

### Study variables

#### Dependent variables – knowledge questions

**Fig. 2:**
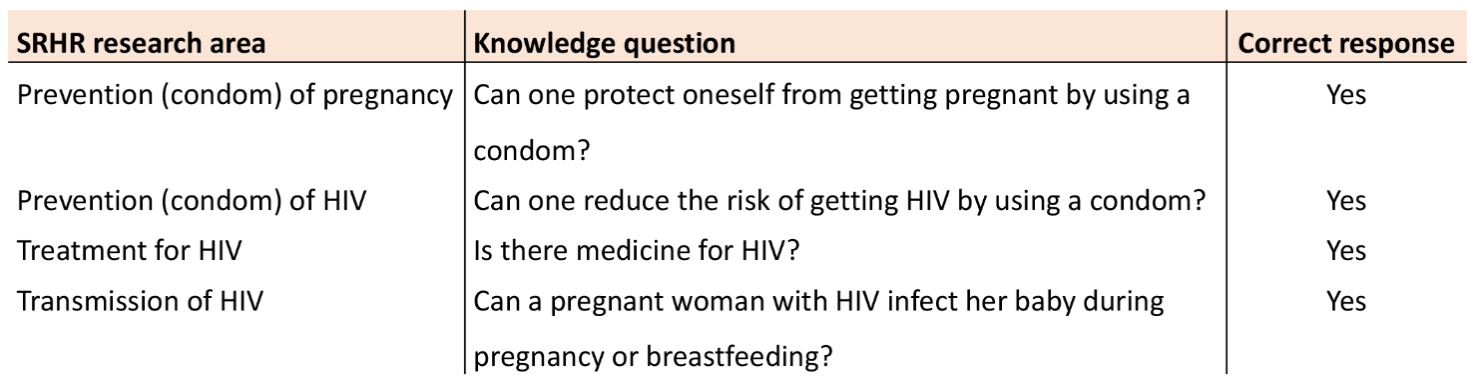
Sexual and reproductive health and rights (SRHR) research areas and questions on the knowledge of condom use for preventing pregnancy and the prevention, treatment and transmission of HIV. The participants’ response choices for all questions were: “yes,” “no,” or “I don’t know.”

The independent variable was the responses as “no or don’t know”, representing incorrect answers to the following questions: (1) Can one protect oneself from getting pregnant by using a condom; (2) Can one reduce the risk of getting HIV by using a condom; (3) Is there medicine for HIV; (4) Can a pregnant woman with HIV infect her baby during pregnancy or breastfeeding? **(**Fig. 2**).** Additionally, we assessed questions related to the perceived need for more SRHR-related information, including where to access contraceptive methods, such as condoms, and where to undergo testing for HIV and other STIs in Sweden.

#### Independent – background variables

The independent variables included in the analyses were age, sex, educational level, religious identity, sexual health education, country region origin, length of time in Sweden, reasons for migration, residence status, and living situation. The age ranged from 15 to 86 years, with a median age of 35 and interquartile range of 20. Age groups were categorised as follows: 15–19 years, 20–24 years, 25–34 years, 35–39 years, and 40 years or older. We focused on younger age groups of migrants and differentiated between those 15–19 years and 20–24 years. These age groups often lack older adults’ resources and life experience and are at higher risk of adverse SRHR outcomes. We dichotomised sex (male, female). We categorised the years of education completed by low level (no school or less than nine years) and high level (10 years or more). We coded having previous sexual health education and no previous sexual health education before arriving in Sweden. Religious identity was coded as Christianity, Islam, other religious identities (including Buddhism and Judaism) and non-religious. The region was classified under the Middle East, North Africa (MENA), South Asia, SSA, and other regions, including European citizens, North, Central, South America, and the Caribbean [33]. Length of time in Sweden was coded as two or more years (long time) and less than one year (short time), based on the year they arrived in Sweden. The reasons for migration were categorised as seeking asylum, family ties, work, study or other reasons, such as European nationals. Residency status in Sweden was also classified as no permit, temporary and permanent residence permit, and European nationals were considered permanent residents. We coded living situations by those living alone, with friends or roommates and partners or family members.

### Statistical analysis

Descriptive statistics were used to summarise the characteristics of the incorrect responses, and the outcomes related to the dependent variable (incorrect responses) through frequencies and percentages. Multivariable regression was conducted to estimate the adjusted odds ratios (aOR) with a 95% confidence interval (CI) for the sociodemographic predictors (age, sex, educational level, religious identity, sexual health education, country region origin, length of time in Sweden, reasons for migration, residence status, and living situation). The outcome variable was the probability of incorrect knowledge of condom use (condoms prevent pregnancy) as Outcome 1, HIV knowledge (prevention as Outcome 2, treatment as Outcome 3, and transmission as Outcome 4). We calculated statistical collinearity to identify instances where assumptions were compromised between the independent variables (sociodemographic predictors). Hypothesis tests at a 5% significance level for the log aOR, considering it equal to zero, were performed using a two-sided Wald-type test based on a standard normal distribution. We conducted descriptive statistics and presented the summative results to describe the perceived need for more SRHR-related information. This includes detailed SRHR-related information on how to obtain condoms, locations where to get STI testing and resources for abortion services or more information on SRHR-related laws in Sweden. Analyses were conducted using Stata/SE 15.1.

### Ethics

We provided detailed information about the study to the participants and collected written informed consent. Participation in the survey was voluntary, and the respondents were not obligated to answer all questions; they could withdraw from the study anytime. The Swedish Ethical Review Authority granted ethical approval (Dnr: 2017/2030-31 and 2018/1002-32).

## Results

Of the 3430 respondents in the survey, nearly 42% reported not knowing that condom use can reduce the risk of HIV infection, and 39% of the respondents did not know that one can protect oneself from getting pregnant by using a condom (Table 1). Most (77%) of the respondents reported that they did not know that treatment medicines exist for HIV, and half (52%) were unaware that a pregnant woman with HIV can transmit the virus to her baby during pregnancy or breastfeeding. Approximately 64% of the respondents indicated a need for more SRHR-related information (Table 2).

**Table 1:** Sociodemographic characteristics of the respondents and knowledge of HIV and condom use in Sweden, 2018

Characteristics	Can one protect oneself from getting pregnant by using a condom? *N* = 3430 (100%)			Can one reduce the risk of getting HIV by using a condom? *N* = 3241 (100%)			Is there treatment for HIV? *N* = 3367 (100%)			Can a pregnant woman with HIV infect her baby during pregnancy or breastfeeding? *N* = 3362 (100%)		
	Correct	Incorrect	*N* (100%)	Correct	Incorrect	*N* (100%)	Correct	Incorrect	*N* (100%)	Correct	Incorrect	*N* (100%)
**Total**	2083 (61%)	1347 (39%)	3430	1878 (58%)	1363 (42%)	3241	763 (23%)	2604 (77%)	3367	1614 (48%)	1748 (52%)	3362
**Age (years)**												
15–19	336 (57%)	259 (43%)	595	281 (50%)	280 (50%)	561	140 (24%)	442 (76%)	582	193 (33%)	394 (67%)	587
20–24	183 (59%)	125 (41%)	308	143 (50%)	140 (50%)	283	54 (18%)	242 (82%)	296	108 (36%)	188 (64%)	296
25–34	536 (64%)	300 (36%)	836	475 (60%)	318 (40%)	793	185 (23%)	634 (77%)	819	437 (54%)	378 (46%)	815
35–39	272 (65%)	146 (35%)	418	240 (60%)	156 (40%)	396	80 (19%)	334 (81%)	414	205 (50%)	207 (50%)	412
40+	558 (61%)	362 (39%)	920	553 (63%)	207 (37%)	760	230 (25%)	683 (75%)	913	505 (55%)	408 (45%)	913
**Sex**												
Female	1119 (58%)	823 (42%)	1942	977 (53%)	856 (47%)	1833	431 (22%)	1477 (78%)	1908	966 (51%)	945 (49%)	1911
Male	941 (65%)	499 (35%)	1440	878 (64%)	485 (36%)	1363	321 (23%)	1091 (77%)	1412	631 (45%)	773 (55%)	1404
**Years of education completed**												
No school	110 (49%)	114 (51%)	224	86 (43%)	144 (57%)	230	46 (22%)	164 (78%)	210	63 (29%)	152 (71%)	215
≤ 9 years of formal school	643 (56%)	495 (44%)	1138	557 (52%)	507 (48%)	1064	227 (20%)	885 (80%)	1112	413 (38%)	688 (62%)	1101
10 to 12 years	600 (60%)	395 (40%)	995	562 (59%)	391 (41%)	953	201 (20%)	784 (80%)	985	533 (54%)	455 (46%)	988
> 12 years	649 (69%)	311 (31%)	960	649 (67%)	315 (33%)	964	269 (27%)	728 (73%)	997	574 (58%)	421 (42%)	995
**Sexual health education**												
Previous sexual health education	1126 (74%)	400 (26%)	1526	1041 (71%)	425 (29%)	1466	453 (30%)	1042 (70%)	1495	860 (58%)	632 (42%)	1492
No previous sexual health education or don’t know	924 (50%)	917 (50%)	1841	828 (47%)	931 (53%)	1759	296 (16%)	1515 (84%)	1811	728 (40%)	1081 (60%)	1809
**Religious identity**												
Christianity	342 (67%)	166 (33%)	508	352 (71%)	143 (29%)	495	154 (31%)	365 (69%)	519	266 (53%)	234 (47%)	500
Islam	963 (58%)	712 (42%)	1675	818 (51%)	788 (49%)	1606	293 (18%)	1361 (82%)	1654	772 (47%)	874 (53%)	1646
Other religious*	371 (66%)	191 (34%)	562	361 (68%)	171 (32%)	532	168 (31%)	377 (69%)	545	250 (46%)	298 (54%)	548
**Native origin region**												
MENA	894 (59%)	635 (41%)	1529	769 (53%)	694 (47%)	1463	245 (16%)	1281 (84%)	1526	841 (55%)	677 (45%)	1518
South Asia	211 (56%)	165 (44%)	376	164 (48%)	178 (52%)	342	62 (17%)	296 (83%)	358	83 (23%)	275 (77%)	358
SSA	332 (64%)	189 (36%)	518	303 (65%)	162 (35%)	465	132 (27%)	362 (73%)	494	229 (47%)	263 (53%)	492
Other region*	563 (66%)	297 (34%)	890	561 (68%)	271 (32%)	832	275 (32%)	574 (68%)	849	413 (48%)	440 (52%)	853
**Year arrived in Sweden**												
2016–2019	1199 (64%)	684 (36%)	1883	1057 (60%)	719 (40%)	1776	455 (25%)	1394 (75%)	1849	971 (53%)	874 (47%)	1845
2013–2015	737 (58%)	529 (42%)	1266	682 (57%)	518 (43%)	1200	247 (20%)	997 (80%)	1244	535 (43%)	709 57%)	1244
2012 or earlier	143 (53%)	129 (47%)	272	132 (52%)	123 (48%)	255	60 (23%)	204 (77%)	264	106 (40%)	157 (60%)	263
**Reason for migration**												
To seek asylum	830 (59%)	569 (41%)	1399	721 (55%)	598 (45%)	1319	243 (18%)	1121 (82%)	1364	639 (47%)	718 (53%)	1357
Family ties	501 (64%)	286 (36%)	787	443 (67%)	200 (33%)	643	100 (31%)	223 (69%)	323	436 (56%)	349 (44%)	785
Work or study	203 (62%)	127 (38%)	330	219 (69%)	98 (31%)	317	194 (25%)	587 (75%)	781	132 (41%)	193 (59%)	325
Other reasons*	260 (57%)	192 (43%)	452	228 (53%)	198 (47%)	426	97 (22%)	346 (78%)	443	191 (43%)	249 (57%)	440
**Resident status in Sweden**												
No residence permit	104 (59%)	73 (41%)	177	95 (59%)	67 (41%)	162	51 (30%)	119 (70%)	170	71 (42%)	98 (58%)	169
Temporary residence permit	689 (64%)	389 (36%)	1078	629 (61%)	394 (39%)	1023	231 (22%)	827 (78%)	1058	518 (49%)	538 (51%)	1056
Permanent residence permit	1142 (60%)	770 (40%)	1912	1011 (56%)	798 (44%)	1809	426 (23%)	1459 (77%)	1885	891 (47%)	991 (53%)	1882
**Living situation**												
Alone	261 (60%)	171 (40%)	432	248 (61%)	161 (39%)	409	97 (23%)	327 (77%)	424	177 (42%)	241 (53%)	418
Friends or multi-residence dwelling	173 (64%)	96 (36%)	269	156 (62%)	94 (38%)	250	58 (22%)	201 (78%)	259	103 (41%)	150 (59%)	253
With partner, relatives, and/or family members	1483 (61%)	945 (39%)	2428	1317 (57%)	986 (43%)	2303	548 (23%)	1843 (77%)	2391	1190 (50%)	1210 (50%)	2400

* Native origin: other regions include North Central, South America, and other European countries. Religious identity: other religious include Buddhism, Hinduism, Judaism or atheism. Reason for migration: other reasons include European nationals

**Table 2:** Sociodemographic characteristics of the respondents’ need for SRHR-related information in Sweden, 2018

Characteristics	Do you need more information about SRHR? * *N* = 3433 (100%)	
	Yes	No
**Total**	2205 (64%)	1228 (36%)
**Age groups (years)**		
15–19	389 (64%)	217 (36%)
20–24	204 (69%)	91 (31%)
25–34	522 (62%)	315 (38%)
35–39	269 (64%)	151 (36%)
40+	564 (62%)	340 (38%)
**Sex**		
Female	1231 (64%)	697 (36%)
Male	939 (65%)	517 (35%)
**Years of education completed**		
No school	155 (70%)	68 (30%)
≤ 9 years of formal school	765 (67%)	378 (33%)
10 to 12 years	639 (64%)	355 (36%)
> 12 years	599 (60%)	408 (40%)
**Sexual health education**		
Previous sexual health education	932 (60%)	619 (40%)
No previous sexual health education or don’t know	1264 (68%)	602 (32%)
**Religious identity**		
Christianity	330 (63%)	193 (37%)
Islam	1117 (65%)	614 (35%)
Other religious*	381 (65%)	203 (35%)
**Native origin region**		
MENA	989 (65%)	530 (35%)
South Asia	256 (67%)	129 (33%)
SSA	342 (65%)	186 (35%)
Other region*	511 (60%)	338 (40%)
**Year arrived in Sweden**		
2016–2019	1173 (62%)	709 (38%)
2013–2015	832 (66%)	433 (34%)
2012 or earlier	193 (70%)	85 (30%)
**Reason for migration**		
To seek asylum	926 (66%)	479 (34%)
Family ties	207 (60%)	137 (40%)
Work or study	485 (64%)	269 (36%)
Other reasons*	302 (68%)	142 (32%)
**Resident status in Sweden**		
No residence permit	101 (58%)	74 (42%)
Temporary residence permit	700 (65%)	372 (35%)
Permanent residence permit	1209 (64%)	699 (36%)
**Living situation**		
Alone	312 (71%)	125 (29%)
Friends or multi-residence dwelling	183 (65%)	99 (35%)
With partner, relatives, and/or family members	1500 (63%)	901 (37%)

*The question on more information about sexual and reproductive health and rights (SRHR) in Sweden included details regarding SRHR laws, abortion services, and access to obtaining contraceptive methods such as condoms, information on sexually transmitted infections (STIs), including testing locations. Native origin: other regions include North Central, South America, and other European countries. Religious identity: other religious include Buddhism, Hinduism, Judaism or atheism. Reason for migration: other reasons include European nationals

The multivariable logistic regression (Table 3) showed that not knowing a condom can prevent pregnancy was associated with the following variables: age groups 15–19 years (aOR: 1.35; 95% CI 1.02–1.79); female respondents (aOR: 1.68; 95% CI 1.36–2.07); low level of education (aOR: 1.30; 95% CI 1.04–1.61); no previous sexual health education (aOR: 2.57; 95% CI 2.11–3.13). Having family ties (aOR: 0.62; 95% CI 0.46–0.85) for migrants as the reason for migration decreased the odds by 38% not knowing condoms can prevent pregnancy compared to migrating to Sweden as a European citizen.

**Table 3:** Multivariable associations with lack of knowledge and HIV prevention, treatment and transmission and sociodemographic characteristics

Outcome	1			2			3			4		
	Do not know condoms prevent pregnancy			Do not know condoms prevent HIV			Do not know HIV treatment exists			Do not know HIV transmission from mother to child		
Sample	*n* = 2025			*n* = 1965			*n* = 1995			*n* = 1992		
	aOR	95% CI	*P* value	aOR	95% CI	*P* value	aOR	95% CI	*P* value	aOR	95% CI	*P* value
**Age**,** years**												
15–19	1.35	1.02–1.79*	0.035	2.05	1.53–2.75*	0.000	0.87	0.63–1.20	0.419	2.26	1.69–3.02*	0.000
20–24	1.21	0.86–1.71	0.269	1.96	1.37–2.80*	0.000	1.53	0.98–2.38	0.056	1.76	1.24–2.50*	0.001
25–34	0.95	0.73-1-23	0.715	1.25	0.96–1.63	0.093	1.16	0.86–1.57	0.312	0.99	0.77–1.28	0.985
35–39	0.92	0.66–1.27	0.629	1.14	0.81–1.59	0.433	1.58	1.06–2.34*	0.022	1.09	0.79–1.49	0.584
40+	Ref											
**Sex**												
Male	Ref											
Female	1.68	1.36–2.07*	0.000	1.95	1.57–2.42*	0.000	1.11	0.87–1.42	0.360	1.00	0.82–1.23	0.933
**Religious identity**												
Christianity	Ref											
Islam	1.14	0.85–1.53	0.357	1.65	1.21–2.23*	0.001	1.49	1.08–2.05*	0.014	1.09	0.82–1.45	0.543
Other religious identity*	0.95	0.66–1.37	0.795	1.19	0.81–1.76	0.363	0.85	0.58–1.23	0.402	1.25	0.88–1.78	0.207
**Education**												
High level (> 10 years)	Ref											
Low level (no school or ≤ 9 years)	1.30	1.04–1.61*	0.017	1.41	1.13–1.77*	0.002	0.89	0.69–1.16	0.410	1.74	1.40–2.15*	0.000
**Sexual health education**												
Previous sexual health education	Ref											
No previous sexual health education	2.57	2.11–3.13*	0.000	2.49	2.04–3.04*	0.000	2.21	1.76–2.77*	0.000	2.23	1.84–2.71*	0.000
**Country region**												
MENA	Ref											
South Asia	0.89	0.63–1.26	0.535	1.11	0.78–1.58	0.552	0.89	0.57–1.39	0.632	3.31	2.23–4.92*	0.000
Sub-Saharan Africa	0.85	0.63–1.15	0.313	0.58	0.42–0.81*	0.001	0.60	0.42–0.85*	0.005	1.14	0.84–1.55	0.377
Other regions (North, Central, South America, and Europe)	0.91	0.69–1.19	0.500	0.66	0.50–0.87*	0.004	0.64	0.47–0.88*	0.006	1.44	1.10–1.88*	0.007
**Length of time in Sweden**												
≥ 2 years	Ref											
< 1 year	0.92	0.72–1.16	0.492	1.08	0.84–1.37	0.531	1.05	0.80–1.38	0.680	0.86	0.68–1.09	0.224
**Reason for migration**												
Other reasons*	Ref											
Asylum seeker	0.86	0.64–1.16	0.341	1.02	0.74–1.39	0.895	1.23	0.86–1.77	0.240	0.71	0.52–0.97*	0.032
Work or study	1.05	0.70–1.56	0.803	0.91	0.59–1.39	0.666	0.97	0.62–1.52	0.909	1.10	0.74–1.65	0.619
Family ties	0.62	0.46–0.85*	0.025	0.80	0.58–1.09	0.172	0.77	0.55–1.09	0.151	0.62	0.45–0.84*	0.002
**Resident status in Sweden**												
No residence permit	Ref											
Temporary	0.85	0.51–1.40	0.526	1.48	0.84–2.60	0.173	1.27	0.73–2.21	0.364	1.39	0.82–2.37	0.215
Permanent	0.92	0.56–1.52	0.764	1.65	0.94–2.89	0.076	1.15	0.67–1.98	0.605	1.40	0.83–2.36	0.203
**Living situation**												
Alone	Ref											
Friends/multi-residence	0.82	0.53–1.26	0.374	0.84	0.54–1.33	0.476	1.08	0.65–1.80	0.750	0.90	0.58–1.41	0.670
Family members	0.88	0.66–1.19	0.435	1.15	0.84–1.57	0.357	1.13	0.80–1.60	0.466	1.11	0.82–1.50	0.477

*P-value significant at < 0.05. Abbreviations: Ref reference; aOR Adjusted odds ratio; CI confidence interval; Middle East and North Africa (MENA); Sub-Saharan Africa (SSA). The multivariable model was adjusted for sociodemographic predictors (age, sex, educational level, religious identity, sexual health education, country region origin, length of time in Sweden, reasons for migration, residence status, and living situation). Religious identity: other religious include Buddhism, Hinduism, Judaism or atheism. Reason for migration: other reasons include European nationals

The following variables were associated with not knowing that condoms can prevent HIV: young age groups 15 and 19 years had an aOR of 2.05 (95% CI 1.53–2.75); 20 and 24 years (aOR: 1.96; 95% CI 1.37–2.80); female respondents (aOR: 1.95; 95% CI 1.57–2.42); identified religious as Islam (aOR: 1.65; 95% CI 1.21–2.23) compared to Christianity; low level of education (aOR: 1.41; 95% CI 1.13–1.77); no previous sexual health education (aOR: 2.49; 95% CI 2.04–3.04). Country origin from other regions, including the Americas and European countries (aOR: 0.66; 95% CI 0.50–0.87), or from the SSA (aOR: 0.58; 95% CI 0.42–0.81), decreased the odds between 34 and 42% not knowing that condoms can prevent HIV compared to the respondents originating from the MENA region.

The variables associated with lack of knowledge that HIV treatment exists included: ages between 35 and 39 years (aOR: 1.58; 95% CI 1.06–2.34); Islam (aOR: 1.49; 95% CI 1.08–2.05) compared to Christianity as religious identity; no previous sexual health education (aOR: 2.21; 95% CI 1.76–2.77). Originating from the other regions (aOR: 0.64; 95% CI 0.47–0.88) and SSA (aOR: 0.60; 95% CI 0.42–0.85) had a 36–40% decreased odd of not knowing about HIV treatment when compared to those originating from the MENA region.

Young respondents, between the ages of 15 and 19 (aOR: 2.26; 95% CI 1.69–3.02); 20 to 24 years (aOR: 1.76; 95% CI 1.24–2.50); a low level of education (aOR: 1.74; 95% CI 1.40–2.15); no previous sexual health education (aOR: 2.23; 95% CI 1.84–2.71); originating from South Asia (aOR: 3.31; 95% CI 2.23–4.92); or from the other regions (aOR: 1.44; 95% CI 1.10–1.88) were strongly associated with not knowing that HIV transmission can occur from mother to child. Family ties (aOR: 0.62; 95% CI 0.45–0.84) for migrants and asylum seekers (aOR: 0.71; 95% CI 0.52–0.97) as the reason for migration to Sweden had 29–38% decreased odds of incorrectly answering the question about mother-to-child transmission of HIV compared to European nationals.

## Discussion

We found that the majority of young migrants, both men and women, had significant knowledge gaps about condoms, HIV treatment, and methods for preventing HIV infection. Specifically, 39% of participants were unaware of condoms in preventing unplanned pregnancies, and only 68% were aware that condoms can reduce the risk of HIV infection. Our analyses revealed that the socio-demographic characteristics of being a woman, young migrant aged 15–24, lacking prior sexual health education, and having limited schooling background were associated with knowledge gaps about condoms preventing pregnancy. Additionally, a lack of knowledge about mother-to-child HIV transmission was associated with having migrated from South Asia or other regions, including the Americas. Over half of the respondents expressed a need for more information related to SRHR. Addressing these knowledge gaps is important for developing effective SRHR-related policies for migrants that align with the SDGs.

Young migrants and those without comprehensive sexual health education, as our analyses revealed, lack awareness of the preventive aspects of condom use against HIV, preventing pregnancy and the potential for HIV transmission from mother to child. We note that these findings, especially considering that the lack of knowledge about condoms’ dual protective role for both pregnancy and HIV, not only impacts reproductive health but also increases vulnerability to HIV. Awareness and understanding that condoms can prevent pregnancy and HIV may be influenced by several aspects, such as cultural beliefs, misconceptions, and the absence of comprehensive sexual health education, both in migrants’ home countries and in their host countries [34]. Research on young first-generation immigrants in Switzerland indicated that a lack of accurate knowledge on HIV transmission and a lack of awareness about HIV, in combination with the sex differences on condom use, may be influenced by the perception of living in a safe environment compared to their home country, thus making them more vulnerable to HIV [35]. Limited access to sexual health education and stigma surrounding open discussions on sexuality and sexual health, coupled with traditional, more conservative gender norms influencing decisions about sexual relations [36, 37], may explain why some women in our study indicated lower knowledge about condom use compared to men. Furthermore, religious identity was found in our analyses to increase the likelihood of incorrect knowledge regarding the prevention of HIV through condom use and treatment for HIV. Certain religious beliefs and values may endorse a positive view towards promoting the prevention and treatment of HIV or discourage the use of condoms and promote abstinence, leading to misconceptions about the effectiveness of condoms in preventing HIV transmission [38, 39].

Our analyses revealed that knowledge gaps about HIV and condom use to prevent pregnancy were particularly pronounced among young migrants aged 15–24. In addition, we found a strong association between age groups 35 to 39 years and a lack of awareness about the existing HIV treatment, especially among those with no previous sexual health education. This association might be attributed to several factors, including limited access to sources of information. For instance, some individuals may continue to hold outdated or inaccurate information about HIV due to this lack of access to current knowledge [40]. Another contributing factor to the lack of HIV knowledge might be the misconception held by some older individuals who mistakenly believe that they are not at risk of contracting HIV, as it has been indicated in other research among older age groups in the United Kingdom [41]. Misconceptions about HIV might lead to not only a decreased likelihood of seeking HIV testing or information, assuming that they are not personally susceptible, but also stigmatisation of people living with HIV because of limited understanding and inaccurate beliefs about available treatments for HIV diagnosis [42].

Conservative societal and cultural gender and sexuality norms contribute to restricting access to sexual health services in certain countries and often hinder comprehensive discussions about SRHR. In regions such as South Asia and other regions, including the Americas, where the prevalence of HIV is lower compared to SSA [43], there may be a reduced emphasis on HIV-related education. Our research revealed that individuals migrating to Sweden for family reasons were more aware of the usefulness of condoms than for reasons such as migrating as quota refugees. A possible explanation is that in Sweden, the civic orientation establishment program offered to all migrants who have been granted residency due to asylum reasons or family reunification includes SRHR information. This may lead to a more supportive environment, fostering information exchange, including discussions on sexual health [44]. For young migrants with family connections and economic stability, this support system could lead to increased awareness of reproductive health matters, such as the efficacy of condoms in preventing pregnancy. Asylum seekers, benefiting from comprehensive support systems and healthcare services in host countries, often undergo health screenings during the asylum process, providing valuable information on various health issues, including preventing mother-to-child transmission of HIV. Our findings align with previous research in Finland, emphasising the period during which an asylum claim is being processed provides an opportunity for health education and HIV testing [26].

### Strengths and limitations

The large and representative sample of new migrants in Sweden provides a representative view of the association between sociodemographic factors and knowledge of preventing pregnancy with condoms and information about HIV. We also collected a large sample size in different languages and targeted a broader range of recently arrived migrants. However, we did not assess migrants’ knowledge of other risk-taking practices or behaviours related to HIV risk or HIV-associated stigma [31]. The subject of HIV, pregnancy or the word ‘condoms’ may be sensitive to individuals, and this may influence their perceptions and responses. In addition, the respondents’ pre-existing perceptions, including attitudes, practices, or beliefs, of condoms and HIV might have shaped their understanding and, consequently, their reported knowledge. There is also a potential response bias concerning the question of HIV treatment. For some respondents, the concept of treatment for an illness might imply that a cure is possible, which is not the case with HIV. Despite these limitations, our findings have implications for future research, including identifying knowledge gaps related to SRHR, especially among older migrants, and reflecting on the need to incorporate and evaluate comprehensive sexual health education or interventions among these groups.

## Conclusion

There are significant SRHR knowledge gaps among migrants in Sweden. The findings strongly suggest that comprehensive sexual health education is necessary for providing adequate information to migrants about unwanted pregnancy, contraceptive methods and preventing STIs after migrating to Sweden. Our results suggest that current sexual and reproductive health education does not effectively reach migrant groups in Sweden. The lack of knowledge about HIV among young individuals regarding their susceptibility to HIV demonstrates the importance of comprehensive sexual health education and outreach efforts. Additionally, limited access to updated information for older migrants highlights the need to ensure accurate and current information reaches all demographics, with particular attention to individuals from diverse cultural backgrounds who may have limited sexual health education.

## Data Availability

The datasets generated and analysed during this study are not publicly available due to ethical approval (Dnr: 2017/2030-31 and 2018/1002-32) and the restrictions that apply to the data availability. The approval specifies that data must uphold participant anonymity, be accessed only in its entirety by the research group and be securely stored with password protection. For data access, please contact the corresponding author corresponding author, Veronika Tirado (email: veronika.tirado@ki.se), who can provide the information upon a reasonable request.

## Abbreviations

AIDS: Acquired Immune Deficiency Syndrome
ART: Antiretroviral therapy
aOR: Adjusted odds ratio
CI: Confidence interval
HIV: Human Immunodeficiency Virus
MENA: Middle East and North Africa
PMTCT: Prevention of mother-to-child transmission
SSA: Sub-Saharan Africa
SRHR: Sexual and reproductive health and rights
STI: Sexually transmitted infections
